# Fracture of Penis: Amputation; Recovery

**Published:** 1898-06

**Authors:** N. B. Smith

**Affiliations:** Basil, Ohio


### FRACTURE OF PENIS; AMPUTATION; RECOVERY.

By N. B. Smith, D.V.M.,
BASIL, OHIO.

In the March issue of your valued Journal I noticed the report of a case of fracture of the penis in a bull. As I had a similar case, I concluded to submit it to your readers, thinking it might be of interest to them.

*Subject.* A five-year-old Jersey bull.

*History.* I know but very little in regard to the history. When the animal was first seen by its owner, the penis was protruding about three inches, with the sheath in a swollen condition. Three days after I was called, this being June 14, 1897. I found the animal in a stable with sheath terribly swollen, with penis hanging down, and gangrene having already set in. I then cast the animal and after an examination found that amputation was the only resort. The penis was so swollen I could neither replace nor withdraw it.

*Operation.* I split the sheath and prepuce open, and then had assistant draw out the penis, which I found broken up, about four inches suppurating, and the whole sheath filled with maggots. To get above the diseased tissue I had to amputate eight inches of the penis, which I did by ligaturing above the seat of operation, in order to prevent hemorrhage. I then dissected out the urethra, leaving it a quarter of an inch longer than where the penis was amputated, to prevent stricture of same when healed. Amputated with knife; ligated the arteries, removed ligature above amputation; cleansed well with carbolized solution; filled the wound with boric acid. I then let the animal up and had him tied up in the stable, and the wound washed daily with carbolized solution, after which he made a complete recovery. While he is of no service whatever as a breeder, yet I think this a remarkable case, considering the length of time the case had gone, the condition I found it in, the extremely hot weather, and the lax way a farmer usually follows your instructions. During the whole period I only made the one visit.

